# The GLP-1 analog, liraglutide prevents the increase of proinflammatory mediators in the hippocampus of male rat pups submitted to maternal perinatal food restriction

**DOI:** 10.1186/s12974-018-1370-7

**Published:** 2018-12-05

**Authors:** Y. Diz-Chaves, L. Toba, J. Fandiño, L. C. González-Matías, L. M. Garcia-Segura, F. Mallo

**Affiliations:** 1 Laboratory of Endocrinology, Biomedical Research Center (CINBIO), University of Vigo, Campus As Lagoas-Marcosende, E-36310 Vigo (Pontevedra), Spain; 2 Instituto Cajal, Consejo Superior de Investigaciones Científicas (CSIC), Avenida Doctor Arce 37, E-28002 Madrid, Spain; 3 Centro de Investigación en Red Fragilidad y Envejecimiento Saludable (CIBERFES), Instituto de Salud Carlos III, Madrid, Spain

**Keywords:** Maternal food restriction, Inflammation, Cytokines, Microglia, Astroglia, Hippocampus, Incretins, GLP-1, Liraglutide

## Abstract

**Background:**

Perinatal maternal malnutrition is related to altered growth of tissues and organs. The nervous system development is very sensitive to environmental insults, being the hippocampus a vulnerable structure, in which altered number of neurons and granular cells has been observed. Moreover, glial cells are also affected, and increased expression of proinflammatory mediators has been observed. We studied the effect of Glucagon-like peptide-1 receptor (GLP-1R) agonists, liraglutide, which have very potent metabolic and neuroprotective effects, in order to ameliorate/prevent the glial alterations present in the hippocampus of the pups from mothers with food restriction during pregnancy and lactation (maternal perinatal food restriction—MPFR).

**Methods:**

Pregnant Sprague-Dawley rats were randomly assigned to 50% food restriction (FR; *n* = 12) or ad libitum controls (CT, *n* = 12) groups at day of pregnancy 12 (GD12). From GD14 to parturition, pregnant FR and CT rats were treated with liraglutide (100 μg/kg) or vehicle. At postnatal day 21 and before weaning, 48 males and 45 females (CT and MPFR) were sacrificed. mRNA expression levels of interleukin-1β (IL1β), interleukin-6 (IL-6), nuclear factor-κβ, major histocompatibility complex-II (MHCII), interleukin 10 (IL10), arginase 1 (Arg1), and transforming growth factor (TGFβ) were assessed in the hippocampus by quantitative real-time polymerase chain reaction. Iba1 and GFAP-immunoreactivity were assessed by immunocytochemistry.

**Results:**

The mRNA expression IL1β, IL6, NF-κB, and MHCII increased in the hippocampus of male but not in female pups from MPFR. In addition, there was an increase in the percentage of GFAP and Iba1-immupositive cells in the dentate gyrus compared to controls, indicating an inflammatory response in the brain. On the other hand, liraglutide treatment prevented the neuroinflammatory process, promoting the production of anti-inflammatory molecules such as IL10, TGFβ, and arginase 1, and decreasing the number and reactivity of microglial cells and astrocytes in the hippocampus of male pups.

**Conclusion:**

Therefore, the GLP-1 analog, liraglutide, emerges as neuroprotective drug that minimizes the harmful effects of maternal food restriction, decreasing neuroinflammation in the hippocampus in a very early stage.

## Background

An optimal maternal nutrient supply has a critical role in placental-fetal growth and development. During pregnancy, maternal malnutrition or undernutrition hampers placentation, with resulting changes in placental size, morphology, and blood flow [1] that thereby reduce the supply of nutrients to the fetus. The subsequently compromised fetal nutrition status may have profound effects in organogenesis, growth, and fetal programming, resulting in intrauterine growth restriction (IUGR) and newborns with low birthweight [1], both associated with short-term and long-term effects in development and increased morbidity [2].

The development of the nervous system is very sensitive to environmental insults, which influence the temporal and regional arising of critical developmental processes (i.e., proliferation, migration, differentiation, synaptogenesis, myelination, and apoptosis) [3]. In this regard, undernutrition during early life causes deficits and distortions of brain structure and function. In particular, the hippocampus is highly affected, altering its morphology, causing a deficit in the total number of dentate gyrus granule cells and neurons, and since a vulnerable structure to undernutrition. [4–6]. Additionally, malnutrition of the mother attenuates the field excitatory postsynaptic potentials, reduces dendritic spine density, and affects morphology of hippocampus, and so it alters learning and memory-based behaviors [7, 8].

However, not just the neurons or the neuronal activities are affected by maternal undernutrition, glial cells, and in particular microglia and astrocytes, are altered too. Different studies have described increased activation of microglia in IUGR brain [9], encompassing a number of processes including increased production of pro-inflammatory cytokines [10], decreased production of anti-inflammatory cytokines [11], and astrogliosis [12, 13] promoted by maternal undernutrition.

Microglial signaling is critical for synaptic development in embryonal period and early postnatal life, while also controlling maturation of functional brain connectivity and complex behavior into adulthood [14]. However, microglia respond to changes in diet [15] and maternal malnutrition exposure coincides with a developmental window of increased microglial-mediated brain patterning, regulating neuronal cell number throughout the brain by initiating normal programmed cell death and engulfing dead or dying cells, increasing the differentiation of neural stem/precursor cells into astrocytes, and modulating synapse numbers as well as synaptic function and maturation [14, 16, 17]. Moreover, while microglia activation normally exerts a protective action on the CNS (central nervous system), its increased chronical activation may become however deleterious [18]. There are two different degrees of microglial activation as similarly described in macrophages. The phenotype of these cells have been viewed as a dichotomy, showing stages M1: generating inflammatory cytotoxic response; and M2: activate in response to stimuli and to promote tissue repair and resolution of inflammation [19–21]. Similar phenotypes have been attributed to microglia in the central nervous system. Importantly, microglial phenotypes reflect expression of cell surface receptors and release of soluble factors with relevant functions [22].

Likewise, immune signaling is crucial for proper brain development. Pro-inflammatory cytokines not only serve as signals for brain development, regulating processes such as cellular differentiation, survival, and axonal guidance [23], but also are secreted in response to cellular injury. The overproduction of pro-inflammatory cytokines, as happening in maternal malnutrition, may promote neuronal damage and losses [9]. Since microglia and cytokine signals may play such a critical role in brain development, that early shifts in these pathways induced by maternal malnutrition have the capacity to change cognitive control and behavior into adulthood [14].

In humans, maternal malnutrition significantly elevates the risk for neurodevelopmental disorders in children. This is especially important for neurodevelopmental disorders such as autism spectrum disorders (ASD), attention-deficit/hyperactivity disorder (ADHD), and schizophrenia [14, 24, 25]. It also promotes significant changes in learning and memory, performance in hippocampus-dependent behavioral test and modifies cognitive behaviors that govern food preference and food intake [14, 26]. In addition, the maternal perinatal undernutrition increases the offspring risks for metabolic dysfunction and diseases, both in humans and in animal models [27, 28].

In this context, it has been described that maternal food restriction during late pregnancy up to weaning induces IUGR, decreases beta-cell mass and beta-cell proliferation [29, 30], and total insulin content respect to controls, what leads to gradual glucose intolerance at 4 months and diabetes at 17 months of age [31, 32]. GLP-1R activation stimulates β-cell proliferation in the pancreas by recruiting stem cell, increases glucose-dependent insulin secretion, and lowers blood glucose in patients with T2D (type 2 diabetes) [33]. Exendin-4 also increases Pdx1 expression in islets promoting b-cell preservation [34] and preventing diabetes in intrauterine growth-retarded (IUGR) animal model [35].

Additionally, GLP-1 receptor agonists have been shown to have neuroprotective, neurotrophic, and anti-inflammatory effects in the central nervous system [36–38]. GLP-1, liraglutide, and other GLP-1 analogs cross the blood-brain barrier [39] where they specifically bind to GLP-1 receptor [40]. GLP-1R is expressed in hypothalamus, hippocampus, and neurons of different brain areas [41]. GLP-1 prevented the lipopolysaccharide (LPS)-induced interleukin 1β (IL-1β) and interleukin-6 (IL-6) mRNA expression in cultured rat astrocytes [42], promotes neurogenesis, neuronal survival, and synaptic integrity; it restores long-term potentiation and ameliorates cognitive deficits [43] and GLP-1R signaling in the ventral hippocampus reduces food preference for high palatable-food [44].

Regarding the neurological alterations of pups induced by food restriction of the mother, testing glucagon-like peptide-1 receptor (GLP-1R) agonists as liraglutide to ameliorate/prevent these alterations, may be a promising possibility. For this purpose, we have administrated the liraglutide to pregnant dams under food restriction during last 8 days of pregnancy, and we have studied the inflammatory response mediators in the hippocampus in early stages of development, when brain dysfunctions have arisen, but cognitive or metabolic alterations induced by undernutrition are not established yet.

## Material and methods

The animal facility at the University of Santiago de Compostela (Spain) provided Sprague-Dawley female (*n* = 24) and male (*n* = 12) rats. The animals were housed at the facility of the University of Vigo in a 12-h light/dark cycle (lights on at 09:00), controlled temperature (20 °C–22 °C), and ad libitum access to water and standard chow (A04; Panlab, Barcelona). Special care was taken to minimize suffering and to reduce the number of animals used to the minimum required for statistical accuracy. The experimental procedures were conducted under the European Union guidelines for the use of animals for experimental purposes (Council Directive 2010/63/EU), and they were approved by the Ethical Committee of the University of Vigo and Xunta de Galicia (protocol register number: ES360570215601/17/ FUN01/FIS02/LCGM/02).

Adult virgin Sprague-Dawley rats (2 months of age) were group-housed (4/cage) to synchronize their estrous cycle. The stage of the estrous cycle was determined by vaginal cytology. Females in estrous were individually housed for 24 h in the presence of a sex-experienced male rat. Females were then examined to detect vaginal plug, which was used to confirm mating. The day of plug detection was regarded as gestational day 1 (GD1). At GD12, pregnant rats were randomly assigned to food restriction [30, 31] (50% of daily intake of control dams. MPFR; *n* = 12) or ad libitum control groups (CT; *n* = 12) and individually housed in plastic breeding cages. From gestational day 14 to parturition, pregnant rats (MPFR and CT) were treated with liraglutide (dose 100 μg/kg/12 h; s.c.; MPFR/LIRA or CT/LIRA; Bachem, Bubendorf) or vehicle (saline and acetic acid 0.4%; s.c.). At parturition, food restriction was decreased to 30% until weaning. Each litter usually contained 10–16 fetuses. Litter size was adjusted to 11 pups per dam, with similar numbers of males and females. To obviate any litter effects, animals used for each experiment were randomly chosen from different litters, and only a limited number of animals (*n* = 1–2) was used from each litter.

At postnatal day 21 (PD21) and before weaning, 48 males and 45 females (control and food-restricted pups) were sacrificed by decapitation and the brain was removed. The left hemisphere was immersed in 4% paraformaldehyde (VWR Chemicals, BDH Prolabo) in 0.1 M phosphate buffer, pH 7.4 during 72 h, and then rinsed with phosphate buffer and stored at − 20 °C in a cryoprotective solution. The hippocampus was dissected from the other half of the brain and stored at − 80 °C for real-time RT-PCR analysis.

### Real-time RT-PCR analysis

Interleukin 1β (IL1β), interleukin 6 (IL6), nuclear factor-κB (NF-κB), major histocompatibility complex-II (MHCII), interleukin 10 (IL10), arginase-1 (ARG1), and transforming growth factor (TGFβ) mRNA levels were assessed in the hippocampus by quantitative real-time polymerase chain reaction. Hippocampi were homogenized and total RNA extracted by using TRI reagent® solution following the manufacturer’s instructions (Ambion, USA). First strand cDNA was prepared from 2 μg RNA using M-MLV reverse transcriptase (Promega, Madison, WI, USA) according to the manufacturer’s protocol. The cDNA was amplified by real-time PCR using a SYBR Green master mix in an ABI Prism 7500 Sequence Detector (AB), with conventional AB cycling parameters (40 cycles of 95 °C, 15 s; 60 °C, 1 min). Primer sequences were designed using Primer Blast (NCBI): IL1β: Forward 5´-TACCTATGTCTTGCCCGTGG-3′, Reverse: 5´-TAGCAGGTCGTCATCATCCC-3′; IL6: Forward: 5´-CTGCTCTGGTCTTCTGGAGT-3, Reverse: ´5-TGGAAGTTGGGG-TAGGAAGG-3′; NF-κB: Forward 5′- CATCCACCTTCATGCTCAGC-3´ Reverse 5´-CCACCACATCTTCCTGCTTG-3′; MHCII: Forward: 5´-TTCCAGCCCCCATGTCAG-3′, Reverse: 5´-ACAACCCCAGGGCACAGA-3′; IL10: Forward: 5´-AGACCCACATGCTCCGAGAG-3′, Reverse 5´-GGGCATCACTTCTACCAGGT-3′; TGFβ: Forward: 5´-CCAGCCGCGGGACTCT-3, Reverse: 5´-TTCCGTTTCACCAGCTCCAT-3′; ARG1: Forward 5´-GCAGAGACCCAGAAG-AATGGAAC-3´ Reverse 5´-CGGAGTGTTGATGTCAGTGTGAGC-3′ 1; 18 s was selected as control housekeeping gene: Forward 5´-CGCCGCTAGAGGTGAAATTCT-3´ Reverse 5´-CATTCTTGGCAAATGCTTTCG-3′. Data were represented using the comparative cycle threshold (Ct) method. To validate a ΔΔCt value, we checked that the amplification efficiency of the target and the reference genes were comparable (the absolute value of the slope of ΔCt vs log relative concentration between − 0.1 and 0.1). The Ct was determined for each target and reference gene in duplicate. Then, ΔΔCt was calculated by normalizing the ΔCt of each sample to the mean ΔCt value of the male CT/VEH group.

### Immunohistochemistry

Sagittal sections of the hippocampus, 50 μm thick, were obtained using a Vibratome (VT 1000S, Leica Microsystems, Wetzlar, Germany). Immunohistochemistry was carried out in free-floating sections under moderate shaking. Sections for all experimental groups were processed in parallel in each assay run to decrease variability. Endogenous peroxidase activity was quenched for 10 min at room temperature in a solution of 3% hydrogen peroxide in 30% methanol. After several washes in 0.1 M phosphate buffer (pH 7.4), containing 0.3% BSA, 0.3% TritonX-100, and 0.9% NaCl (washing buffer), sections were incubated overnight at 4 °C with one of the following primary antibodies: a rabbit polyclonal antibody to Iba1 (Ionized calcium binding adaptor molecule 1, a marker of microglia) corresponding to C-terminus (Wako Chemical Industries, Japan; diluted 1:2000), a rabbit polyclonal antibody to glial fibrillary acidic protein (GFAP, a marker of astroglia; Dako Cytomation, DK-2600, Glostrup, Denmark Z-0334, diluted 1:1000). Primary antibody was diluted in washing buffer containing 3% normal goat serum. After incubation with the primary antibody, sections were rinsed in buffer and incubated for 2 h at room temperature with biotinylated goat anti-rabbit immunoglobulin G (Pierce Antibody; Rockford, IL 61101, USA; diluted 1:300 in washing buffer). After several washes in buffer, sections were incubated for 90 min at room temperature with avidin–biotin peroxidase complex (ImmunoPure ABC peroxidase staining kit, Pierce). The reaction product was revealed by incubating the sections with 2 μg/ml 3,30-diaminobenzidine (Sigma–Aldrich) and 0.01% hydrogen peroxide in 0.1 M phosphate buffer. Then, sections were dehydrated, mounted on gelatinized slides, and examined with a Nikon NiE microscope (Nikon Instruments Europe B.V).

### Morphometric analysis

The morphometric analysis was randomly performed single blind by a researcher that was unaware of the identity of the experimental groups. The number of Iba1- and GFAP-immunoreactive cells was estimated with the optical dissector method in the hilus of dentate gyrus of hippocampus, using total section thickness for dissector height at 40× [45, 46] and a counting frame of 220 × 220 μm. A total of 42 counting frames (7 × 6 sections) were assessed/animal for Iba immunostaining and 30 (5 counting frames ×  6 sections) for GFAP immunostaining. In addition, the percentage of Iba1 immunoreactive cells with different morphologies was also assessed only in males. To this aim, for each animal, 120 cells were analyzed in the hilus of the dentate gyrus. Cells were classified in five morphological types as previously described [47]: type I, cells with few cellular processes; type II, cells showing 3–5 short branches; type III cells with numerous (> 5) and longer cell processes, and a small cell body; type IV, cells with large somas and retracted and thicker processes and type V, cells with amoeboid cell body, numerous short processes and intense Iba1 immunostaining. Types IV and V were considered as reactive morphologies, and their sum was represented as the percentage of total cells as was previously described [48].

### Statistical analysis

Data are presented as mean ± SEM. Statistical analyses were performed using GraphPad Prism6 software (GraphPad Software, San Diego, CA, USA). Main and interactive effects were analyzed by two-way analysis of variance (ANOVA) for factorial measures. When appropriated and justified by the ANOVA analysis, differences between group means were analyzed by the Tukey’s post hoc test. Differences were statistically significant following conventions at *p* ≤ 0.05.

## Results

### Food restriction during pregnancy decreased body weight gain of the dams independently of liraglutide

**Fig. 1:**
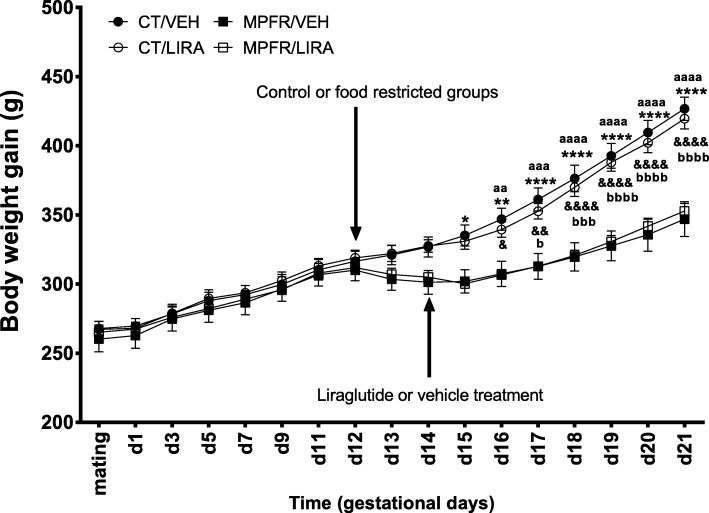
Body weight gain of pregnant dams throughout the food restriction protocol, from mating until day 21 of gestation. Pregnant rats were randomly submitted to food restriction (50% of daily intake of control dams/MPFR) from gestational day 12 or were fed ad libitum in the control group (CT; see arrow). From gestational day 14 to parturition, dams were treated with liraglutide (LIRA; 100 μg/kg/12 h, see arrow) or vehicle. Data are mean + SEM. (MPFR/VEH compared to CT/VEH: *p < 0.05, **p < 0.01, ****p < 0.0001; MPFR/VEH compared to CT/LIRA: ^&^*p* < 0.05, ^&&^*p* < 0.01, ^&&&&^*p* < 0.0001; MPFR/LIRA compared to CT/VEH: ^aa^*p* < 0.01, ^aaa^*p* < 0.001, ^aaaa^*p* < 0.0001; MPFR/LIRA compared to CT/LIRA: ^bbb^*p* < 0.001, ^bbbb^*p* < 0.0001)

After beginning on day 12 of gestation the food restriction protocol (50% of daily intake of control dams), a significantly decrease in the body weight gain was observed from day 14 of pregnancy in food-restricted dams compared to controls fed ad libitum (MPFR/VEH, *n* = 6; CT/VEH; *n* = 6). The treatment with liraglutide from day 14 of gestation until delivery did not revert this decrease in body weight in food-restricted dams, but delayed 1 day the beginning of significantly body weight decrease (MPFR/LIRA; *n* = 6) and did not affect body weight gain in controls (CT/LIRA; *n* = 6). Repeated measures two-way ANOVA showed a significant effect of time (Fig. 1; *F*_(16,320)_ = 640.7; *p* < 0.0001), treatment (*F*_(3,20)_ = 4.97; *p* = 0.0097), subjects (matching; *F*_(20,320)_ = 115.3; *p* < 0.0001), and interaction between the different variables (*F*_(48,320)_ = 29.06; *p* < 0.0001; differences between MPFR/VEH and CT/VEH groups were observed on day 14 (*p* < 0.05), day 15 (*p* < 0.01) and from day 16 until day 21 (*p* < 0.0001); differences between MPFR/VEH and CT/LIRA groups were observed on day 17 (*p* < 0.05), day 18 (*p* < 0.001), and from day 19 until day 21 (*p* < 0.0001); differences between MPFR/LIRA and CT/VEH were observed on day 15 (*p* < 0.01), day 16 (*p* < 0.001), and from day 17 until day 21 (*p* < 0.0001); differences between MPFR/LIRA and CT/LIRA groups were observed on day 17 (*p* < 0.05), day 18 (*p* < 0.001), and from day 19 until 21 (*p* < 0.0001); Tukey’s multiple comparisons post hoc test).

### Effect of MPRF in offspring body weight at 21 days of age: interaction with liraglutide prenatal treatment

**Fig. 2:**
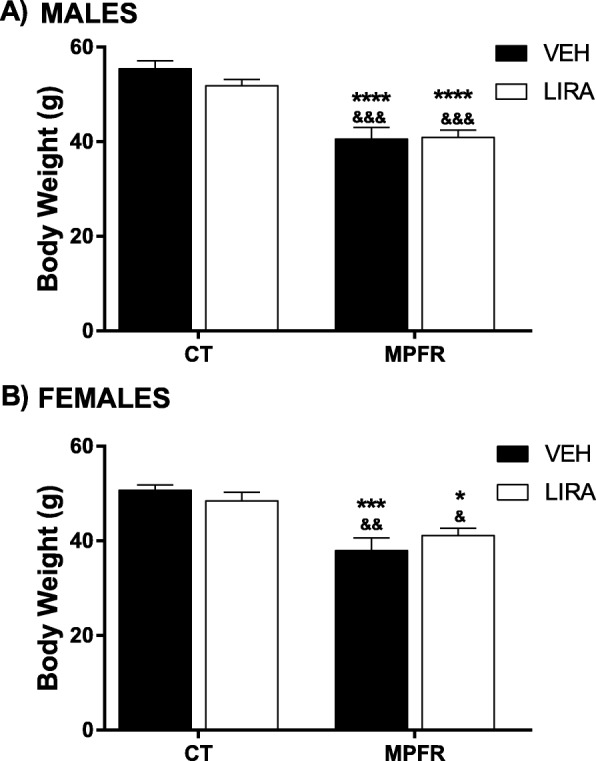
**a** Body weight of male pups at PD21. **b** Body weight of female pups at PD21. CT/VEH, control pups treated with vehicle; CT/LIRA, control pups treated with liraglutide; MPFR/VEH, food-restricted pups treated with vehicle; MPFR/LIRA, food-restricted pups treated with liraglutide. Data are mean + SEM. (*p < 0.05 compared to CT/VEH; ***p < 0.001 compared to CT/VEH; ****p < 0.0001 compared to CT/VEH; ^&^*p* < 0.05 compared to CT/LIRA; ^&&^*p* < 0.01 compared to CT/LIRA; ^&&&^*p* < 0.001 compared to CT/LIRA)

Maternal perinatal food restriction (MPFR/VEH) decreased significantly the body weight of male offspring (*n* = 13; 40.6 ± 2.44 g) at 21 days of age compared to offspring of mothers fed ad libitum during pregnancy and treated with vehicle (CT/VEH; *n* = 11; 55.42 ± 1.66 g) or with liraglutide (CT/LIRA; *n* = 13; 51.85 ± 1.32 g; Fig. 2a). The treatment of food-restricted mothers with liraglutide (MPFR/LIRA) did not revert the body weight decrease of male offspring (*n* = 11; 40.92 ± 1.54). Two-way ANOVA analysis showed a significant diet effect, but not treatment effect nor interaction of both factors (Fig. 2a; *F*_(1,44)_ = 49.21; *p* < 0.0001).

In addition, MPFR decreased significantly body weight of female pups treated with vehicle (*n* = 12; 37.94 ± 2.69 g) compared to controls (CT) treated with vehicle (*n* = 9; 50.7 ± 1.15 g) or liraglutide (*n* = 12; 48.45 ± 1.81 g). The treatment with liraglutide, neither restored body weight in female pups induced by MPFR (*n* = 12; 41.15 ± 1.41 g). Two-way ANOVA analysis showed a significant diet effect, without treatment effect nor interaction (Fig. 2b; *F*_(1,41)_ = 25.57; *p* < 0.0001).

In respect to other parameters of pregnancy, there were no significant effects of food restriction of mothers or the treatment with liraglutide in gestational length, litter size, or sex ratio (Table 1).

**Table 1:** Gestational parameters

Gestational parameter	CT/VEH	CT/LIRA	FR/VEH	FR\LIRA	ANOVA (*F* value)	*p*
Gestational length (days)	22.8 ± 0.3	22.5 ± 0.3	22.9 ± 0.1	22.7 ± 0.2	0,4584	NS
Litter size (number of pups)	14.9 ± 1.3	13.8 ± 0.7	14.8 ± 0.5	15.3 ± 1.3	0,5956	NS
Sex ratio (%, M/(M + F)*100)	48.0 ± 4.7	46.6 ± 4.0	51.8 ± 1.4	49.2 ± 4.4	0,4957	NS

### MPFR increased the number and reactivity of Iba-1 immunopositive cells in the dentate gyrus of males, but not in females: reverted by liraglutide

**Fig. 3:**
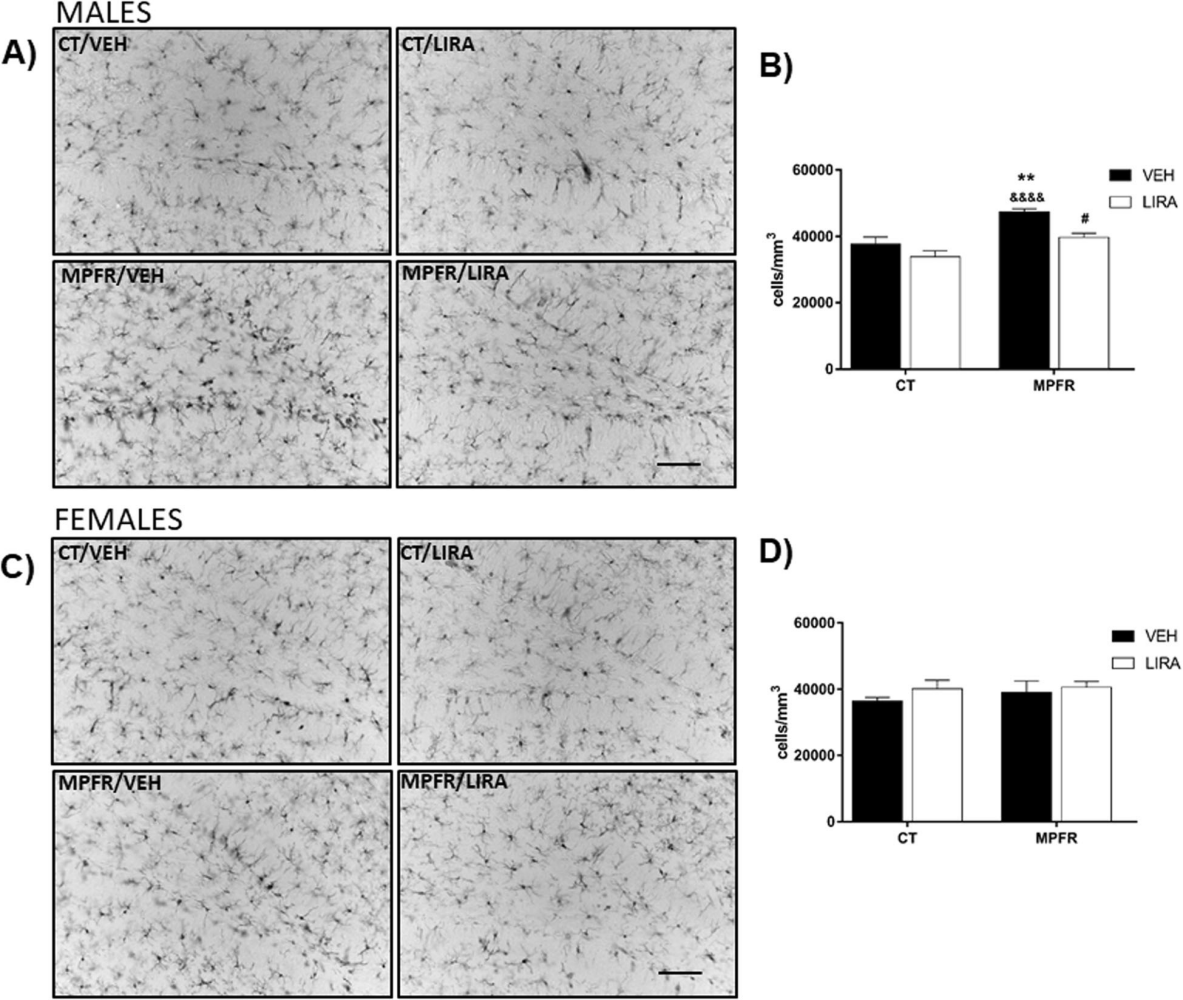
**a** Representative images of Iba-immunoreactivity in the dentate gyrus of male pups. Scale bar, 20 μm. **b** Quantification of Iba-1 immunoreactivity in the dentate gyrus in male pups. **c** Representative images of Iba-immunoreactivity in the dentate gyrus of female pups. Scale bar, 45 μm. **d** Quantification of Iba-1 immunoreactivity in the dentate gyrus in female pups. CT/VEH, control pups treated with vehicle; CT/LIRA, control pups treated with liraglutide; MPFR/VEH, food-restricted pups treated with vehicle; MPFR/LIRA, food-restricted pups treated with liraglutide. Data are mean + SEM. (**p < 0.01 compared to CT/VEH; ***p < 0.001 compared to CT/VEH; ^&&^*p* < 0.01 compared to CT/LIRA; ^&&&&^*p* < 0.0001 compared to CT/LIRA; ^#^*p* < 0.05 compared to MPFR/VEH; ^##^*p* < 0.01 compared to MPFR/VEH)

Male offspring from MPFR treated with vehicle showed increased number of Iba-1-immunopositive cells in the hilus of the dentate gyrus (*n* = 7; 47,298 ± 927.4 cells/mm^3^) compared to male offspring of mothers fed ad libitum (CT) treated with vehicle (*n* = 6; 37,736 ± 2066 cells/mm^3^; Fig. 3a, b) or with liraglutide (*n* = 6; 33,844 ± 1834 cells/mm^3^, Fig. 3a, b). The treatment of mothers fed ad libitum (CT) with liraglutide did not affect the number of Iba-1 immunoreactive cells in male offspring. However, the treatment of food-restricted mothers (MPFR) with liraglutide decreased significantly the number of Iba-1 immunoreactive cells in males (*n* = 5; 39,717 ± 1234 cells/mm^3^, Fig. 3a, b). Two-way ANOVA analysis revealed a diet and treatment effects, without interaction effect (Fig. 3a, b; *F*_(1,20)_ = 23.54; *p* < 0.0001 and *F*_(1,20)_ = 13.18; *p* = 0.0017, respectively; MPFR/VEH vs. CT/VEH *p* < 0.001; MPFR/VEH vs. CR/LIRA, *p* < 0.0001; MPFR/VEH vs. MPFR/LIRA *p* < 0.05). In contrast, maternal food restriction during pregnancy did not affect the number of Iba-1 immunoreactive cells in female offspring, independently of the treatment (Fig. 3c, d, CT/VEH, *n* = 5; 36,463 ± 1048 cells/mm^3^; CT/LIRA; *n* = 5, 40,166 ± 2597 cells/mm^3^; MPFR/VEH, *n* = 5; 39,043 ± 3466 cells/mm^3^; MPFR/LIRA, *n* = 4; 40,631 ± 1735 cells/mm^3^).

**Fig. 4:**
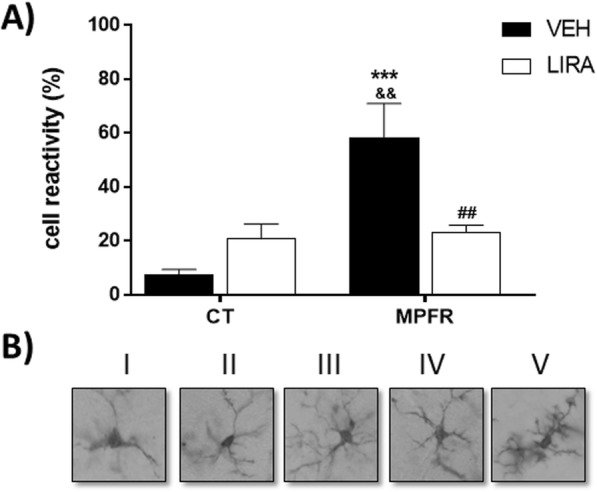
Morphological changes of Iba-1 immunoreactive cells in the hilus of dentate gyrus of the hippocampus. **a** The upper panel show examples of the morphological types in which cells were classified. Type I, cells with few cellular processes; type II, cells showing four short branches; type III, cells with numerous cell processes and a small cell body; type IV, cells with large somas and retracted and thicker processes; and type V, cells with numerous short processes and intense Iba1 immunostaining. **b** The graph shows the proportion of reactive cells (types IV and V) in CT/VEH, control pups treated with vehicle; CT/LIRA, control pups treated with liraglutide; MPFR/VEH, food-restricted pups treated with vehicle; and MPFR/LIRA, food-restricted pups treated with liraglutide. Data are mean + SEM. (***p < 0.001 compared to CT/VEH; ^&&^*p* < 0.01 compared to CT/LIRA; ^##^*p* < 0.01 compared to MPFR/VEH)

Since male pups displayed differences in the number of Iba-1-immunopositive cells between groups, the phenotype of these cells were studied only in males. The percentage of Iba1-immunoreactive cells with larger somas and retracted and thicker processes, a phenotype characteristic of reactive microglia, was increased in male offspring of food-restricted mothers (MPFR) treated with vehicle (Fig. 4b; *n* = 4; 58.09 ± 12.86%) compared to controls treated with vehicle (Fig. 4b *n* = 6; 7.18 ± 2.23%) or liraglutide (Fig. 4b; *n* = 5; 20.71 ± 56%) The treatment of food-restricted mothers (MPFR) with liraglutide decreased significantly the percentage of microglial cells with reactive phenotype (Fig. 4b; *n* = 5; 23.17 ± 2.62%). The two-way ANOVA analysis showed an effect of treatment (Fig. 4b; *F*_(1,16)_ = 19.29; *p* = 0.0005), but not a diet effect. However, an interaction between treatment and diet was observed (*F*_(1,16)_ = 15.89; *p* = 0.0011; MPFR/VEH vs. CT/VEH *p* = 0.0001; MPFR/VEH vs. CT/LIRA *p* = 0.0038; MPFR/LIRA vs. MPFR/VEH *p* = 0.0067, Tukey’s multiple comparisons post hoc test).

### MPFR increased GFAP-immunopositive cells in the dentate gyrus of male, but not in female, offspring: reverted by liraglutide

**Fig. 5:**
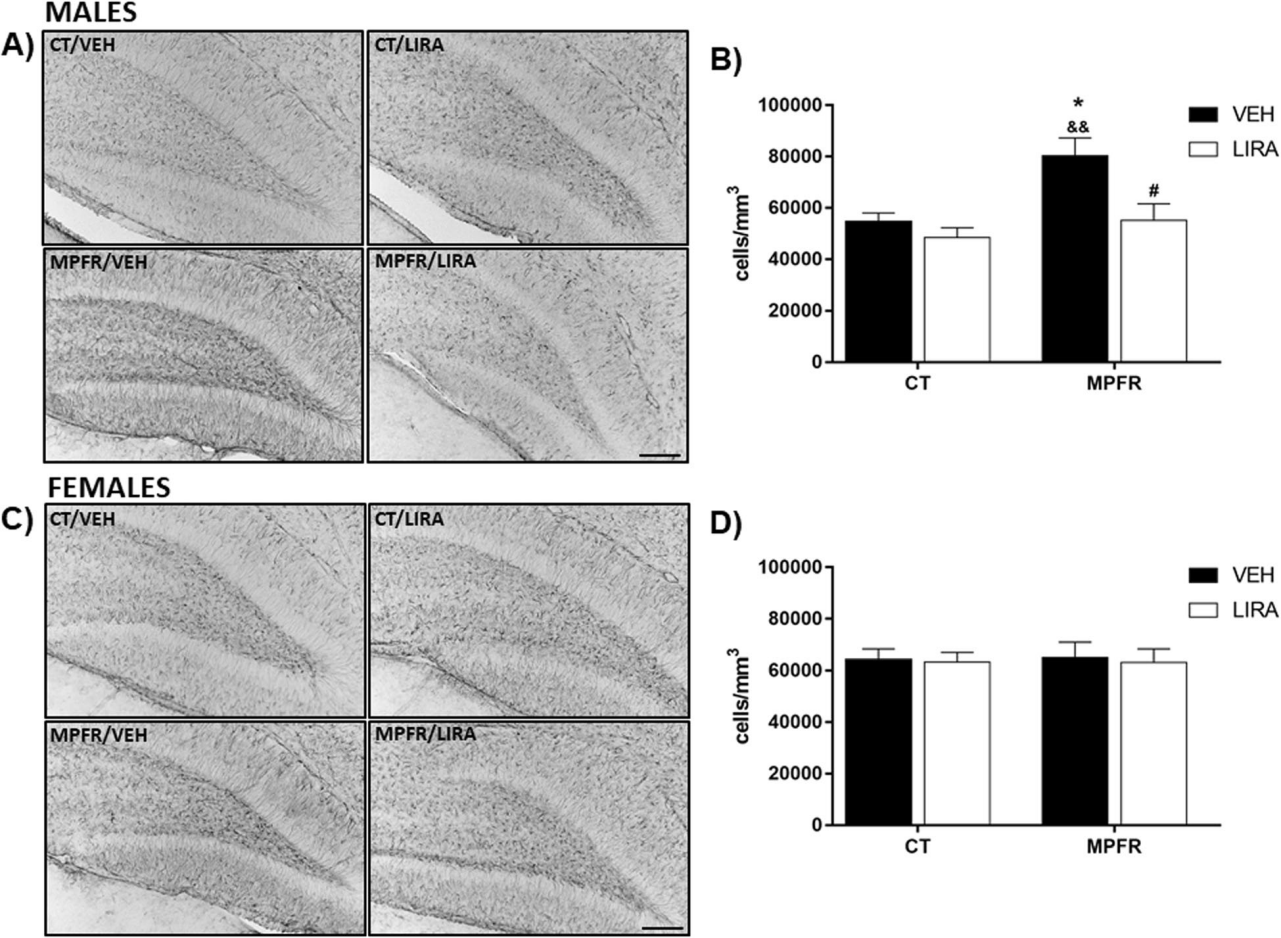
**a** Representative images of GFAP-immunoreactivity in the dentate gyrus of male pups. Scale bar, 30 μm. **b** Quantification of GFAP-1 immunoreactivity in the dentate gyrus in male pups. **c** Representative images of GFAP-immunoreactivity in the dentate gyrus of female pups. Scale bar, 45 μm. **d** Quantification of GFAP-1 immunoreactivity in the dentate gyrus in female pups. CT/VEH, control pups treated with vehicle; CT/LIRA, control pups treated with liraglutide; MPFR/VEH, food-restricted pups treated with vehicle; and MPFR/LIRA, food-restricted pups treated with liraglutide. Data are mean + SEM. (*p < 0.05 compared to CT/VEH; ^&^*p* < 0.05 compared to CT/LIRA; ^#^*p* < 0.05 compared to MPFR/VEH)

The number of GFAP-immunopositive cells was increased in the hilus of the dentate gyrus of male offspring of food-restricted mothers (MPFR) treated with vehicle (Fig. 5a, b; *n* = 4; 80,357 ± 6968 cell/mm^3^), compared to male offspring of control mothers treated with vehicle (*n* = 6; 56,767 ± 3042 cell/mm^3^) or with liraglutide (*n* = 6; 55,119 ± 6469 cell/mm^3^). This effect was not detected in female offspring (Fig. 5c, d; CT/VEH, *n* = 5, 64,349 ± 3903; CT/LIRA, *n* = 5, 63,397 ± 3587; MPFR/VEH, *n* = 6, 65,048 ± 5923 cell/mm^3^). The treatment of food-restricted mothers (MPFR) with liraglutide decreased significantly the number of GFAP immunoreactive cells in male (*n* = 6, 55,119 ± 6469 cells/ mm^3^) but not in female (*n* = 4, 63,183 ± 5092 cells/ mm^3^) offspring. The two-way ANOVA analysis showed a treatment effect (Fig. 5b; *F*_(1,18)_ = 9.2; *p* = 0.0075) and an effect of diet (Fig. 5b; *F*_(1,18)_ = 9.568; *p* = 0.0063; MPFR/VEH vs. CT/VEH *p* < 0.05; MPFR/VEH vs. CT/LIRA *p* < 0.01, and MPFR/VEH vs. MPFR/LIRA *p* < 0.05; Tukey’s multiple comparisons post hoc test), but not an interaction between treatment and diet.

### MPFR increased mRNA of pro-inflammatory and decreased the expression of anti-inflammatory mediators in the hippocampi of male offspring: reverted by liraglutide

**Fig. 6:**
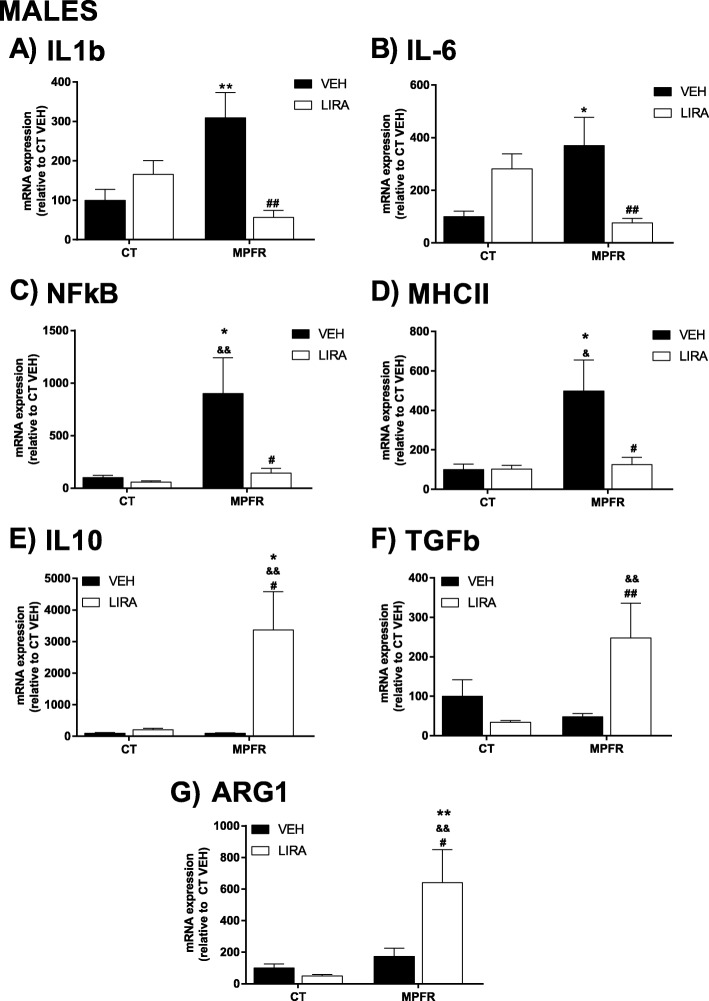
Proinflammatory mediators in male offspring. **a** Interleukin-1b (IL1b). **b** Interleukin-6 (IL-6). **c** Nuclear factor-κB (NF-κB). **d** Major histocompatibility complex-II (MHCII). **e** Interleukin-10 (IL10). **f** Transforming growth factor β (TGFβ). **g** Arginase 1 (ARG1) mRNA levels in the hippocampus. CT/VEH, control pups treated with vehicle; CT/LIRA, control pups treated with liraglutide; MPFR/VEH, food-restricted pups treated with vehicle; MPFR/LIRA, food-restricted pups treated with liraglutide. Data are mean + SEM. (*p < 0.05 compared to CT/VEH; **p < 0.01 compared to CT/VEH; ^&^*p* < 0.05 compared to CT/LIRA; ^&&^*p* < 0.01 compared to CT/LIRA; ^#^*p* < 0.05 compared to MPFR/VEH; ^##^*p* < 0.01 compared to MPFR/VEH)

Maternal perinatal food restriction (MPFR) increased the mRNA expression levels of IL1β in the hippocampi of male offspring (Fig. 6a; *n* = 10; 309.2 ± 64.03), compared to male offspring of mothers fed ad libitum during pregnancy (Fig. 6a; CT/VEH; *n* = 10; 100 ± 27.89) and treated with vehicle. The treatment with liraglutide reverted the effect of maternal food restriction on IL1β mRNA levels in male offspring (Fig. 6a; MPFR/LIRA: *n* = 9; 56.74 ± 17.8), but did not have any effect on males from mothers fed ad libitum (Fig. 6a; *n* = 12; 56.74 ± 17.8). Two-way ANOVA analysis showed a treatment effect (Fig. 6a; *F*_(1,38)_ = 5.245; *p* = 0.0277), not diet effect, but an interaction between treatment and diet effect (Fig. 6a; *F*_(1,38)_ = 15.18; *p* = 0.0004; MPFR/VEH vs. CT/VEH, *p* < 0.01, MPFR/VEH vs. MPFR/LIRA, *p* < 0.001; Tukey’s multiple comparisons post hoc test).

Maternal perinatal food restriction (MPFR) increased the mRNA levels of IL6 in the hippocampi of male offspring (Fig. 6b; *n* = 9; 370.2 ± 107.8), compared to male offspring of mothers feed ad libitum during pregnancy (Fig. 6b; *n* = 11; 100 ± 20.6). The treatment with liraglutide decreased IL6 mRNA levels in males from food-restricted mothers (MPFR/LIRA; Fig. 6b; males; *n* = 10; 75.7 ± 16.8) without effect on IL6 mRNA expression in males from mothers fed ad libitum during pregnancy. The statistical analysis showed not direct effect of diet or treatment, but an interaction between diet and treatment (two-way ANOVA, Fig. 6b; *F*_(1,38)_ = 16.8; *p* = 0.0002; MPFR/VEH vs. CT/VEH, *p* < 0.05, MPFR/VEH vs. MPFR/LIRA, *p* < 0.01, Tukey’s multiple comparisons post-hoc test).

Maternal perinatal food restriction (MPFR) increased mRNA expression of the transcription factor NF-κB in the hippocampi of male offspring (Fig. 6c; *n* = 10; 901 ± 338), compared to males from mothers fed ad libitum (Fig. 6c; CT/VEH; *n* = 9; 100 ± 22.3). The treatment of food-restricted mothers with liraglutide decreased NF-κB mRNA expression compared to the offspring of food-restricted mothers treated with vehicle (Fig. 6c; MPFR/LIRA, *n* = 10; 143.2 ± 45.8). The two-way ANOVA analysis showed a treatment effect (Fig. 6c; *F*_(1,36)_ = 5.41; *p* = 0.025), a diet effect (Fig. 6c; *F*_(1,36)_ = 6.64; *p* = 0.014), and interaction effect between diet and treatment (Fig. 6c; *F*_(1,36)_ = 4.34; *p* = 0.04; MPFR/VEH vs. CT/VEH, *p* < 0.05, MPFR/VEH vs. CT/LIRA, *p* < 0.01, MPFR/LIRA vs. MPFR/VEH, *p* < 0.05; Tukey’s multiple comparisons post hoc test).

Maternal perinatal food restriction increased the mRNA levels of the surface protein MHCII in the hippocampi of male offspring (Fig. 6d; *n* = 10; 498.3 ± 338.), compared to male offspring of mothers fed ad libitum during pregnancy (Fig. 6d; CT/VEH, *n* = 7; 100 ± 27.6). The treatment of food-restricted mothers with liraglutide (MPFR/LIRA) decreased the mRNA levels of MHCII only in males (Fig. 6d; *n* = 9; 126 ± 36.1). The two-way ANOVA analysis revealed a diet effect (Fig. 6d; *F*_(1,32)_ = 5.24; *p* = 0.028) but not a treatment effect. However, an interaction between diet and treatment was observed (Fig. 6d; *F*_(1,32)_ = 4.149; *p* = 0.05; MPFR/VEH vs CT/VEH, *p* < 0.05; MPFR/VEH vs MPFR/LIRA, *p* < 0.05 and MPFR/VEH vs MPFR/LIRA. *p* < 0.05; Tukey’s multiple comparisons post hoc test).

Maternal perinatal food restriction (MPFR) did not significantly modify the mRNA levels of IL-10, transforming growth factor 1 (TGFβ1) and arginase-1 (ARG1) in the hippocampi of male offspring, compared to males from mothers fed ad libitum during pregnancy (Fig. 6e–g). Treatment of control mothers with liraglutide (CT/LIRA) did not affect the mRNA levels of IL-10, TGβ1, and ARG1 in the hippocampi of males (Fig. 6e–g).

In contrast, treatment of food-restricted mothers with liraglutide (MPFR/LIRA) increased the mRNA levels of IL-10 in the hippocampi of males (*n* = 8; 3366 ± 1216) compared to offspring of food-restricted mothers treated with vehicle (Fig. 6e; *n* = 6; 97.8 ± 15.6) or males from control mothers treated with vehicle (Fig. 6e; *n* = 5; 100 ± 21.8) or treated with liraglutide (Fig. 6e; *n* = 10; 209 ± 46.4). The statistical two-way ANOVA analysis showed a diet effect (Fig. 6e; *F*_(1,25)_ = 5.06; *p* = 0.033), a treatment effect (Fig. 6e; *F*_(1,25)_ = 5.8; *p* = 0.023), and an interaction between diet and treatment (Fig. 6e; *F*_(1,25)_ = 5.076; *p* = 0.033; MPFR/LIRA vs. CT/VEH, *p* < 0.05; MPFR/LIRA vs. CT/LIRA, *p* < 0.01; and MPFR/LIRA vs. MPFR/VEH, *p* < 0.05; Tukey’s multiple comparisons post hoc test).

Treatment of food-restricted mothers with liraglutide (MPFR/LIRA) also increased the mRNA levels of TGFβ1 in the hippocampi of male offspring (Fig. 6f; *n* = 9; 247.4 ± 88.7), compared to males from food-restricted mothers treated with vehicle (Fig. 6f; *n* = 13; 47.94 ± 8.31) or from control mothers-treated with liraglutide (Fig. 6f; *n* = 13; 33.8 ± 4.6). The two-way ANOVA analysis showed nor diet or treatment effect, but an interaction between diet and treatment effect (Fig. 6f; *F*_(1,40)_ = 10.65; *p* = 0.0023; MPFR/LIRA vs. CT/LIRA, *p* < 0.01; MPFR/LIRA vs. MPFR/VEH, *p* < 0.01; Tukey’s multiple comparisons post hoc test).

Finally, treatment of food-restricted mothers with liraglutide increased the mRNA levels of ARG1 in the hippocampi of male offspring (Fig. 6g; MPFR/LIRA, *n* = 10, 640.1 ± 209.4), compared to males from food-restricted mothers treated with vehicle (Fig. 6g; MPFR/VEH, *n* = 11; 173 ± 53.1) or males from control-fed mothers treated with vehicle (Fig. 6g; CT/VEH, *n* = 10; 100 ± 26.1) or treated with liraglutide (Fig. 6g; CT/LIRA, *n* = 11; 49.5 ± 10.2). The two-way ANOVA analysis showed a diet effect (Fig. 6g; *F*_(1,38)_ = 10.12; *p* = 0.0029) and an interaction effect between diet and treatment (Fig. 6g; *F*_(1,38)_ = 6.157; *p* = 0.0176; MPFR/LIRA vs. CT/VEH, *p* < 0.01; MPFR/LIRA vs. CT/LIRA, *p* < 0.01, and MPFR/LIRA vs. MPFR/VEH, *p* < 0.05, Tukey’s multiple comparisons post hoc test), but not treatment effect.

**Fig. 7:**
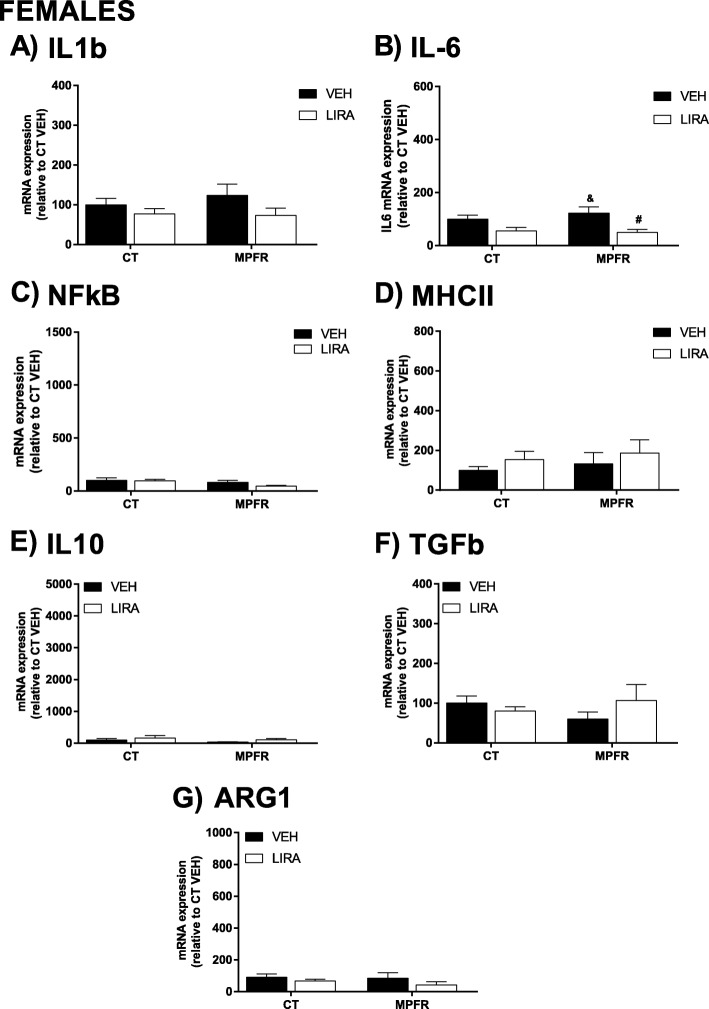
Proinflammatory mediators in female offspring. **a** Interleukin-1b (IL1b). **b** Interleukin-6 (IL-6). **c** Nuclear factor-κB (NF-κB). **d** Major histocompatibility complex-II (MHCII). **e** Interleukin-10 (IL10). **f** Transforming growth factor β (TGFβ). **g** Arginase 1 (ARG1) mRNA levels in the hippocampus. CT/VEH, control pups treated with vehicle; CT/LIRA, control pups treated with liraglutide; MPFR/VEH, food-restricted pups treated with vehicle; MPFR/LIRA, food-restricted pups treated with liraglutide. Data are mean + SEM. (^&^*p* < 0.05 compared to CT/LIRA; ^**#**^*p* < 0.05 compared to MPFR/VEH)

Maternal perinatal food restriction (MPFR) did not increase the mRNA expression levels of IL1β in the hippocampi of female offspring compared to females from mothers treated with vehicle (Fig. 7a; MPFR/VEH: *n* = 12; 123.8 ± 27.87 vs. CT/VEH: *n* = 9; 100 ± 15.92). The treatment with liraglutide did not affect IL1β mRNA expression in females from MPFR or fed ad libitum mothers (Fig. 7a; MPFR/LIRA, *n* = 12; 73.42 ± 17.92 and CT/LIRA, *n* = 12; 76.71 ± 13.69).

Maternal perinatal food restriction (MPFR) did not increase the mRNA expression levels of IL6 in the hippocampi of female offspring compared to females from mothers treated with vehicle (Fig. 7b; MPFR/VEH: *n* = 12; 122.2 ± 24.25 vs. CT/VEH: *n* = 9; 100 ± 14.79). However, the treatment with liraglutide decreased IL6 levels in females from food-restricted mothers (Fig. 7b; MPFR/LIRA; *n* = 10; 49.92 ± 10.87). Two-way ANOVA analysis showed a treatment effect (Fig.7b; *F*_(1,38)_ = 10.98; *p* = 0.002; MPFR/VEH vs. CT/LIRA, *p* < 0.05 and MPFR/LIRA vs. MPFR/VEH, *p* < 0.05), but not diet or interaction effects.

No effect of maternal perinatal food restriction was observed in the mRNA expression of NFκB (Fig. 7 c; MPFR/VEH, *n* = 13; 80.7 ± 18.4; CT/VEH, *n* = 9; 100 ± 25.5). Neither after the treatment with liraglutide of food-restricted or control dams (Fig. 7c; MPFR/LIRA, *n* = 9; 45.15 ± 9.47; CT/LIRA, *n* = 8; 97.4 ± 11.66).

No effect of maternal perinatal food restriction was observed in the mRNA expression of MHCII (Fig. 7d; MPFR/VEH, *n* = 13; 132.7 ± 56.7; CT/VEH, *n* = 7; 100 ± 18.15). Neither after the treatment with liraglutide of food-restricted nor control dams (Fig. 7d; MPFR/LIRA, *n* = 9; 186.2 ± 66.98; CT/LIRA, *n* = 11; 153.7 ± 41.46).

Maternal perinatal food restriction (MPFR) did not significantly modify the mRNA levels of IL-10, transforming growth factor 1 (TGFβ1) and arginase-1 (ARG1) in the hippocampi of female offspring, compared to offspring of mothers fed ad libitum during pregnancy (Fig. 7e; MPFR/VEH, *n* = 12; 39.7 ± 8.6; CT/VEH, *n* = 7; 100 ± 45.61; Fig. 7f; MPFR/VEH, *n* = 12; 59.78 ± 18.18; CT/VEH, *n* = 8; 100 ± 18.06 and Fig. 7g; MPFR/VEH, *n* = 11; 85.12 ± 34.69; CT/VEH, *n* = 10; 100 ± 20.89). The treatment of mothers fed ad libitum during pregnancy with liraglutide (CT/LIRA) or food-restricted dams did not significantly affect the mRNA levels of IL-10, TGβ1, and ARG1 in the hippocampi of female offspring (Fig. 7e; MPFR/LIRA, *n* = 7; 109.8 ± 35.54; CT/LIRA, *n* = 10; 164.5 ± 76.59; Fig. 7f; MPFR/LIRA, *n* = 10; 106.4 ± 40.86; CT/LIRA, *n* = 6; 80.3 ± 11.02 and Fig. 7g; MPFR/LIRA, *n* = 9; 42.39 ± 19.76; CT/LIRA, *n* = 9; 66.71 ± 12.13).

## Discussion

Our study shows that food restriction of the mother increases expression of pro-inflammatory mediators and the number and reactivity of microglia cells and the number of astrocytes in the hippocampus, as early as 21 days of postnatal life. This result shows a clear sex effect, being male pups affected but females do not. The treatment of food-restricted pregnant dams with the GLP-1 receptor analog, liraglutide, was able to prevent this neuroinflammatory response, promoting the production of anti-inflammatory molecules and decreasing the number and reactivity of microglial cells and astrocytes.

Maternal food restriction may affect many brain areas, but the hippocampus is a very vulnerable one. The morphology of hippocampus is altered in response to maternal food restrictions, and the total number of neurons and granule cells are markedly decreased [4–6]. Those structural changes drive to the attenuation of field excitatory postsynaptic potentials and also altered learning and memory-based behaviors [7, 8].

In this study, we have observed an increased number of Iba-1 and GFAP-immunopositive cells in the dentate gyrus of the hippocampus in PD21 males from food-restricted mothers. Our data are in agreement with recent studies in another experimental models of perinatal stress showing increased numbers of activated microglia and astrogliosis, which are suggestive of inflammatory responses in the brain [9]. Also, it has been described that maternal specific protein restriction induced transient microglial activation following birth [49].

It is well known that microglial morphology and the expression of cytokines and chemokines shift dramatically throughout development and it is highly dependent upon sex, having special relevance the activity of androgens during critical periods [50]. In this regard, prenatal insults are associated with increased neurodevelopmental disease risk and impact males significantly more than females [51]. This impact may be reflected by different metabolic and hormonal factors. For example, long-term alterations in central corticotropin-releasing factor (CRF) and glucocorticoid receptor (GR) expression, as well as increased HPA axis responsivity, were present just in male mice offspring exposed to stress early in gestation. Changes in CRF and GR gene methylation correlated with altered gene expression, providing important evidence of epigenetic programming during early prenatal stress [52]. Also examination of placental epigenetic machinery revealed sex differences [52, 53]. In mice, female placental tissue has higher levels of global DNA methylation compared to male’s placentas, which likely provide females additional protection from dynamic changes in gene expression promoted by environmental insults [54]. Furthermore, rodent brain development begins during gestation but mostly continues postnatally. It is well established that the quality and quantity of maternal cares influence epigenetic processes in the developing brain [55]. In this context, we have observed a sex dimorphism in the effect of MPFR on Iba-1 and GFAP-immunopositive cell numbers, since males showed increased reactivity compared to females.

Interestingly, liraglutide treatment was able to decrease the number of Iba-1-immunopositive cells in the dentate gyrus of the hippocampus of males, accordingly with its anti-inflammatory properties, like reducing Iba-1 levels in APP/PS1-mice or irradiated mice [56, 57]. The GLP-1 receptor activation alleviates the chronic inflammation response and mitochondrial stress induced by status epilepticus in the lithium-pilocarpine animal model [54], and also has neuroprotective action modifying the microglia phenotype, increasing microglia complexity (enlarged Feret’s diameter), and improving the number of cell junctions and processes in mice [58]. The GLP-1r is G-protein-coupled receptor which activation induces the start of the AMPc pathway, which in turn activates AMPK and PI3K, promoting several metabolic effects in the target cells [59, 60]. Blocking PKA or PI3K by inhibitors abolished the protective effects of GLP1 in the brain [59]. Whether one or other of these secondary message pathways is the major mediator of the GLP-1 effects at hippocampus would be the matter of specific studies.

The GLP-1 receptor is expressed in both astrocytes and microglia, and GLP-1 and liraglutide induced morphological changes in microglia [42, 57] and astrocytes [57]. We here show that the GLP-1 receptor agonist liraglutide was able to reduce the percentage of Iba1-immunopositive cells with reactive morphology in the dentate gyrus of the hippocampus in males from MPFR. Accordingly, liraglutide also reduced the number of GFAP-immunopositive cells in those animals, similarly to liraglutide effects described in irradiated mice [57].

In this study, we have observed an increased expression of proinflammatory cytokines, such as interleukin-1β (IL-1β) in males but not in females from food-restricted mothers. We and others have previously reported the increased expression of IL-1β in the hippocampus in other animal models of perinatal stress [48, 61]. Activated microglia are the major source of IL-1β in the central nervous system [9]. IL-1β gene expression and protein production are elevated during various disease states, in which this interleukin orchestrates the inflammatory response to various immune stimuli [62]. Through the bound to its receptor IL-1R1, IL-1β has direct effects in glial cells, neurons and endothelial cells, and in immune cells that infiltrate the brain during injury, markedly contributing to local inflammation [63]. IL-1β affects astrocytes the most of all glial cells promoting proliferation, a process known as astrogliosis [64]. Also in microglial cells, it results in the induction of expression and release of several mediators, most of which have neurotoxic actions [63]. Interestingly, liraglutide decreased the mRNA expression of this pro-inflammatory cytokine, IL-1β, in male pups from food-restricted mothers. This effect was previously reported for GLP-1 treatment in cultures of astrocytes and microglia treated with LPS [42]. GLP-1 also decreased the expression of MHCII molecules in the surface of activated microglia, accordingly with the diminution in the percentage of reactive Iba-1-immunopositive cells in the dentate gyrus of the hippocampus.

The stimulation of the classical IL-1β-mediated signaling pathways induces the activation of MAPKs and NF-κB, and release of secondary different mediators (such as IL-6) [63]. Accordingly, we have observed an increased mRNA expression of NF-κB and IL-6 in the hippocampi of males but not in females from food-restricted mothers, suggesting the activation of the pro-inflammatory pathways. The treatment with liraglutide was able to markedly decrease this pro-inflammatory profile. These effects of liraglutide were also described for NF-κB in other cells and tissues such as endothelial progenitor cells and endothelial cells treated with high glucose in vitro [65], in the kidney of diabetic rats [66] and for IL-6 and NF-κB in the adipose tissue of *ob/ob* mice [67]. Likewise, the maternal food restriction programs the adipocyte hypertrophy and/or hyperplasia in the offspring [29]. Adipose explant from rats underwent 50% of subnutrition throughout gestation and then fed with high-fat diet until day 160, showing enhanced secretion and gene expression of TNFα and IL-1β. They also showed a significant increase in gene expression of NLRP3, a key component of the inflammasome complex that regulates the release of IL-1β [30]. These alterations related to reduced circulating insulin was also associated with brain-reduced insulin signaling (IRS2, pAkt, Glut4) while activating pGSK-3βSer9 at 21 days of postnatal life [35]. Overexpression of GSK-3β in transgenic mice induces learning deficits and some features associated with Alzheimer’s disease [36].

Remarkably, we have observed that liraglutide promoted the increased expression of anti-inflammatory mediators, IL-10 and TGFβ1, in the hippocampi of males but not in females from food-restricted mothers. It is well known that microglial cells are able of producing anti-inflammatory cytokines such as IL-10 and transforming growth factor-β1 (TGFβ1), which have neuroprotective effects as it was observed in experimental animal models of traumatic injury and stroke [68, 69]. Signaling through the IL-10 receptor regulates several steps of the immune response, from decreasing cytokine gene expression such as tumor necrosis factor (TNF), IL-1β, IL-6, IL-8, IL-12, and IL-23 [70] to downregulating the expression of major histocompatibility complex class II (MHC-II) [71], and IL-10 induces the differentiation of anti-inflammatory macrophage phenotypes (M2) [70]. Moreover, TGFβ1 promotes a strong anti-inflammatory phenotype in microglia through attenuated cytokine, chemokine, adhesion molecule, and reactive oxidative species (ROS) production [72, 73]. Moreover, the expression of arginase-1 (Arg1) is also increased in males from food-restricted mothers after the treatment with liraglutide. Arg1 is a recognized marker for M2 macrophage/microglia activation that participates in arginine metabolism [74]. Arginase activity by increasing polyamine formation is known to have a positive role in neuroprotection and neural regeneration [75].

The treatment with liraglutide of pregnant dams did not modify the body weight loss caused by food restriction. Moreover, it is interesting to notice, that the observed anti-inflammatory effects were found in a very early stage, at 21 days of postnatal life, when pups still had decreased body weight, both males and females. Accordingly, previous studies in our laboratory have shown no significant effect of liraglutide in the body weight of fetus at E21 from 30% food-restricted mothers compared with that of food-restricted mothers treated with vehicle or between control dams (vehicle or LIRA treated, data not shown).

Numerous studies have established that several different perinatal and postnatal manipulations can program hypothalamic–pituitary–adrenal (HPA) axis function in adult animals [76]. However, the phenotype of HPA function following early manipulation depends on several parameters such as the animal species, the timing and intensity of the stress, the nature of the stressor, and the gender of the fetus or neonate [77]. Any insult is thought to have its most severe impact during the period of rapid brain “growth” that differs greatly among species [78]. During this period, changes induced by undernutrition may become permanent and could perturb numerous brain developmental events as was observed in humans and animal models [79, 80]. Starvation and FR stimulate HPA axis activity in both humans and rats [78]. Furthermore, 50% restricted diet in dams during gestational period has both short- and long-term effects on the HPA axis in male rat offspring [77]. In addition, inappropriate feedback in the HPA axis may play a role in the development of metabolic and cognitive disorders [81]. In this regard, the way by which maternal undernutrition programs the offspring HPA it is not well understood. The involvement of epigenetic mechanism elicited by HPA activation should be taken into account once alterations in gene expression via changes in DNA methylation and/or histone post-translational modifications has been described [77]. Thus, at least in part, some of the effects induced by food restriction might be attributed to alterations in the activation levels of the HPA axis. Complementary, the reduced availability of nutrients during the pregnancy has a direct impact in fetus and also in the placenta, which both undergo metabolic adaptations. Those metabolic adaptations may underlie some of the permanent changes observed in the offspring [82].

In addition, metabolic condition of mothers is associated with higher incidence of some neurodevelopmental diseases (see the Background section, [14, 24, 25]). Once liraglutide is already approved for the treatment of obesity [83], not just in T2D, a window of new therapeutic options becomes open. In spite of liraglutide treatment during pregnancy in humans was not systematically studied still, there is the case of a T2D mother exposed to liraglutide during the first trimester showing not any developmental abnormalities in the child she is carrying [84]. In this regard, our result provides evidence that some benefits might brought up to neurodevelopment of fetus, by different mechanisms which include anti-inflammatory effects. Moreover, in Parkinson’s disease, the efficacy and safety of liraglutide is being investigated in a phase II trial and also for the treatment of Alzheimer’s disease [83]. Those studies in adults support the neuroprotective role of incretin analogs in neurodegenerative diseases by having potent anti-inflammatory actions [37]. Altogether, our and other’s results suggest that liraglutide may improve trophic conditions of neural tissue undergoing development.

In order to describe in detail and go deeper in the mechanism underlying the neuroprotective actions of liraglutide, further studies for evaluating synaptic function, morphological changes in neurons and viability, morphological characterization of microglia, or behavioral changes would be needed, as not addressed in the present study (Fig. 6).

**Fig. 8:**
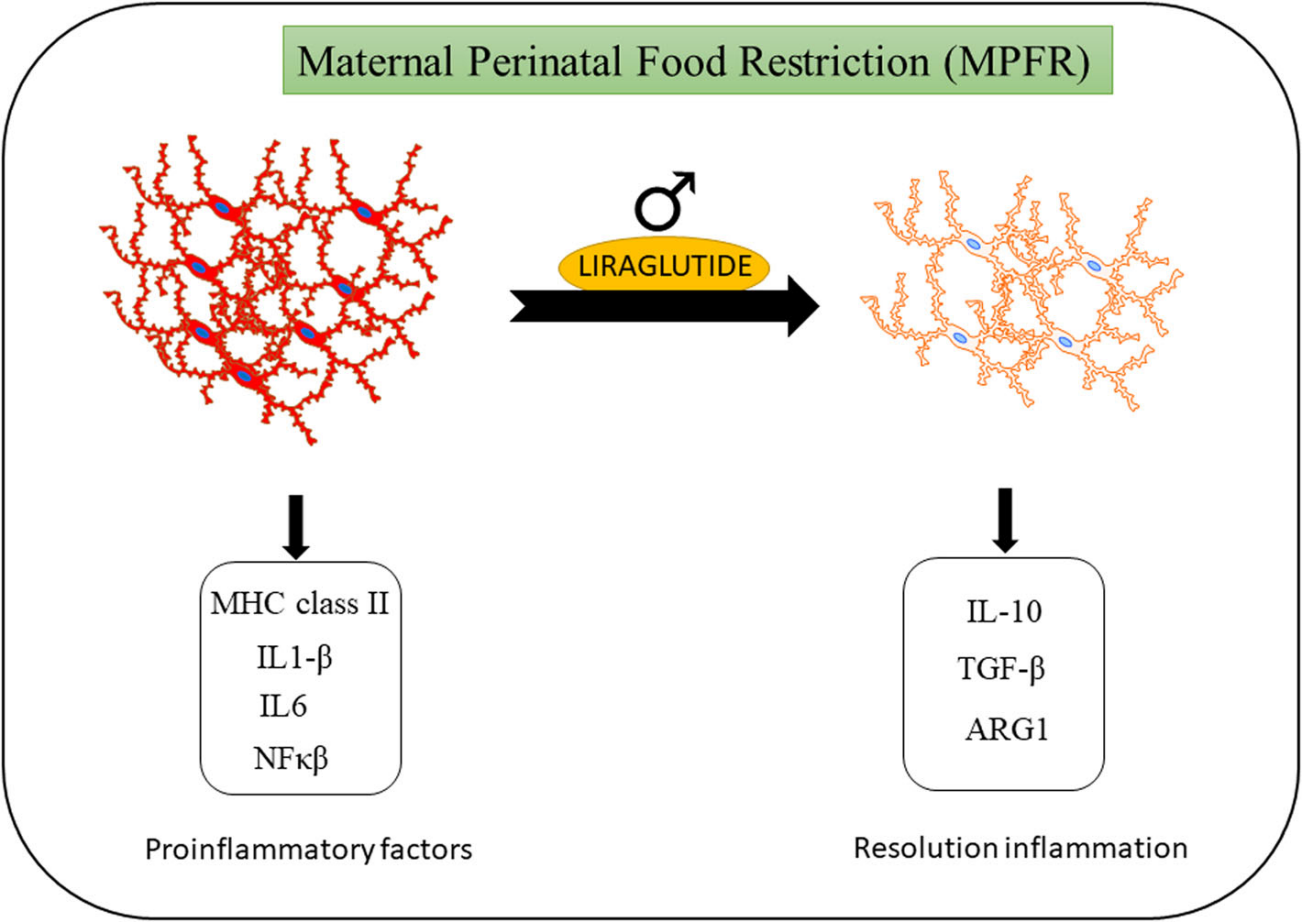
Increased number of Iba-1-immunopositive cells (microglia) was observed in the maternal perinatal food restriction (MPFR) model. Increased mRNA expression of proinflammatory factors: major histocompatibility complex II (MHC type II) molecules, Interleukin-1β (IL-1β), interleukin-6 (IL-6), and the transcription factor NF-κB. The administration of liraglutide to malnourished pregnant rats promoted a protective microglial phenotype in the litters, decreasing the reactivity and number of Iba-1-immunopositive cells (microglia), able of producing protective and anti-inflammatory factors: interleukin-10 (IL-10), transforming growth factor-β (TGF-β) or arginase 1 (Arg1). These effects were just observed in males

In summary, our findings revealed that perinatal food restriction of the mother have clear effects in brain inflammation at early stages of postnatal life, increasing the number and reactivity of Iba-1-immunopositive cells and also increasing the number of GFAP-immunopositive cells. The treatment with liraglutide of food-restricted mothers reduced neuroinflammation not only by decreasing the expression of pro-inflammatory cytokines, but also by decreasing the number and reactivity of microglial cells indicated by Iba-1-immunopositive cells and the number astrocytes related to GFAP-immunopositive cells (Fig. 8).

## Conclusions

Therefore, the GLP-1 analog, liraglutide, emerges as potential neuroprotective drug that minimizes the harmful effects of maternal food restriction, decreasing neuroinflammation in the hippocampus by both reducing the local expression of pro-inflammatory cytokines (IL-1β) and increasing the expression of anti-inflammatory mediators (IL-10 and TGFβ1) at very early stages and before any metabolic alteration might be established.

## Abbreviations

FR: Food restriction
GFAP: Glial fibrillary acidic protein
HPA: Hypothalamo-pituitary-adrenal
Iba1: Ionized calcium binding adaptor molecule
IL-10: Interleukin-10
IL-12: Interleukin-12
IL1β: Interleukin-1beta
IL-23: Interleukin-23
IL-6: Interleukin-6
IL-8: Interleukin-8
MPFR: Maternal perinatal food restriction
T2D: Type 2 diabetes
TGFβ: Transforming growth factor-β
